# Supplementary material for: Homologous bd oxidases share the same architecture but differ in mechanism
Source: Nat Commun. 2019 Nov 13;10:5138. doi: 10.1038/s41467-019-13122-4 (PMC6853902; doi:10.1038/s41467-019-13122-4)
Supplement: Supplementary file 1 — Supplementary Information [file 41467_2019_13122_MOESM1_ESM.pdf]

Supplementary Information

Homologous *bd* oxidases share the same architecture but differ in mechanism

Alexander Theßeling, Tim Rasmussen et al.

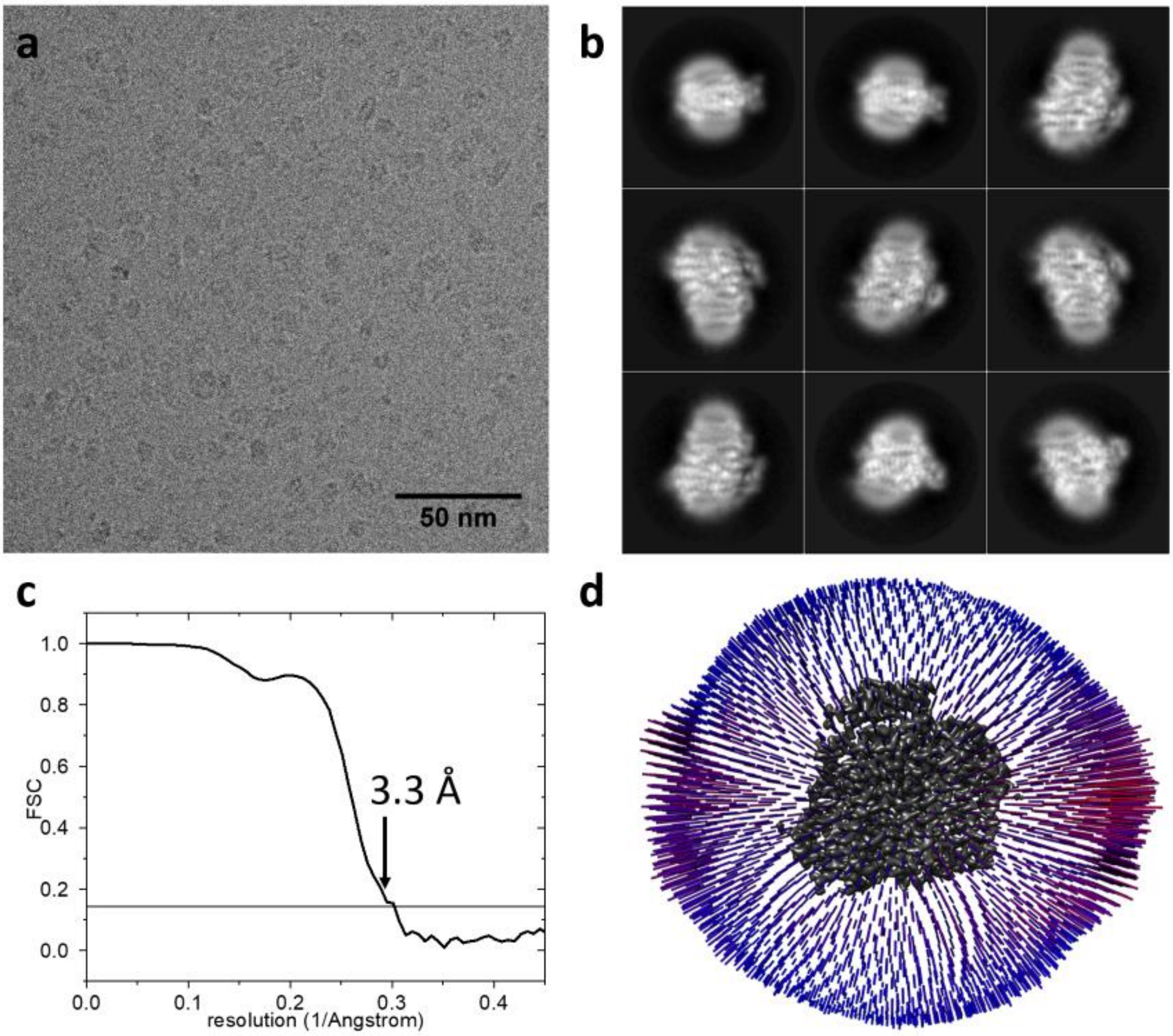

Supplementary Figure 1 | Cryo-electron microscopy on the *bd* oxidase. (a) cryo-EM micrograph of *bd* oxidase. (b) examples of 2D classes of the *bd* oxidase. The edge of each image corresponds to 150 pixels (159.6 Å). (c) Fourier Shell Correlation plot. The overall resolution was determined according to the „gold standard“ from independently processed half-sets to 3.3 Å as implemented in Relion. (d) Angular distribution of particles contributing to the final reconstruction.

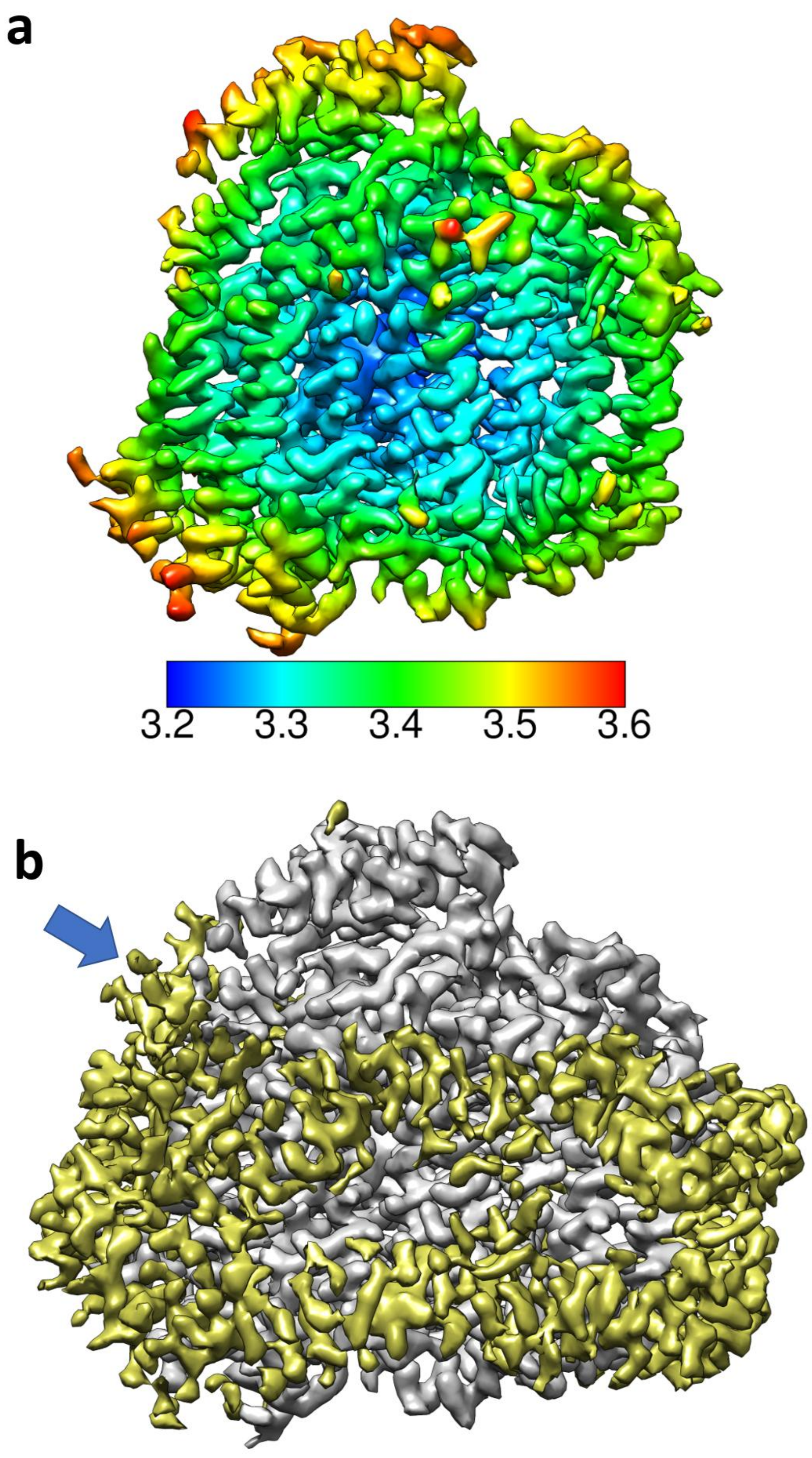

Supplementary Figure 2 | Local resolution and membrane localisation. (a) Local resolution (in Å) of *bd* oxidase map determined with the locres tool in relion postprocess. (b) The noise belt (yellow) is seen at a lower threshold and was not modelled. It is caused by lipids and amphipol molecules and indicates the attachment region of the membrane. In addition, the unresolved Q-loop causes noise (arrow).

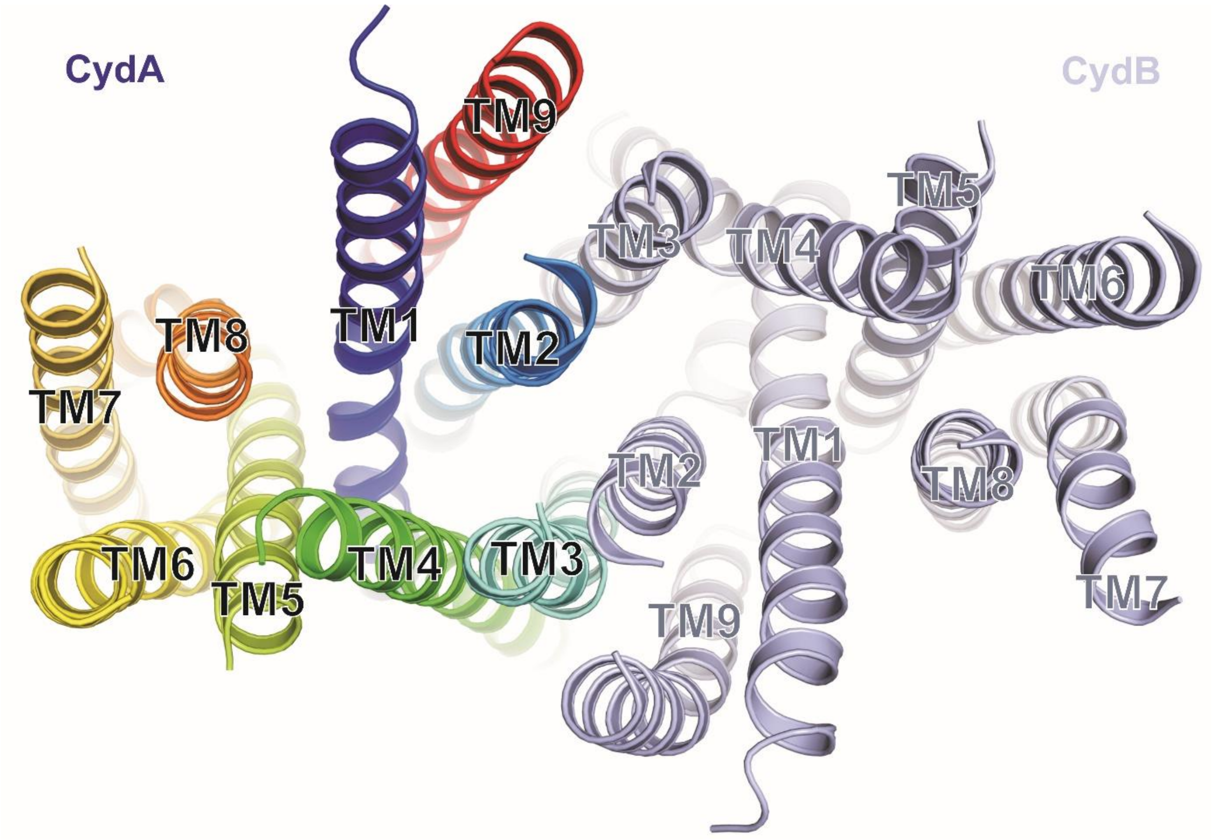

Supplementary Figure 3 | Transmembrane architecture of *E. coli bd* oxidase. View from periplasmic side. Transmembrane helices are assigned consecutive numbers TM1-TM9. Note, periplasmic loops have been removed here for clearer illustration. CydA is given in rainbow colors from N-terminus (dark blue) to C-terminus (red), while CydB is shown in light blue.

A) Alignment of CydA

```
Ec         1  --MLDIVELS RLQFALTAMY HFLFVPLTLG MAFLLAIMET VYVLSGKQIY KDMTKFWGKL FGINFALGVA 68
Gt         1  MNGYDPVLLS RILTELTLTV HIIYATIGVG VPLMIAIAQW VGIRKNDMHY ILLARRWTRG FVITVAVGVV 70
                  * * ** *:   **    *:::..: :* :.:::** :  * : ...  *   ::: * :  * *..*:**.

Ec        69  TGLTMEFQFG TNWSYYSHYV GDIFGAPLAI EGLMAFFLES TFVGLFFFGW DRLG-KVQHM CVTWLVALGS 137
Gt        71  TGTAIGLQLS LLWPNFMQLA GQVISLPLFM E-TFAFFFEA IFLGIYLYTW DRFENQKKHL LLLIPVAIGS 139
              ** :: :*:.   *. : : . *:::. ** : *  :***:*:  *:*:::: * **:  : :*:  :   **:**

Ec       138  NLSALWILVA NGWMQNPIAS DFNFETMRME MVSFSELVLN PVAQVKFVHT VASGYVTGAM FILGISAWYM 207
Gt       140  SASAMFITMV NAFMNTPQG- -FELKNGELV NIDPIVAMFN PAMPTKVAHV LATSYMTSAF VLASIAAWHL 207
              . **::* :. *.:*:.* .   *:::. .:   :.    ::* *.  .*..*. :*:.*:*.*: .: .*:**::

                                                          |L|              Q-loop
Ec       208  LKGRDFAFAK RSFAIAASFG MAAVLSVIVL GDESGYEMGD VQKTKLAAIE AEWETQPAPA AFTLFGIPDQ 277
Gt       208  WKGNRHIYHR KALHLTMKTA FIFSVASALV GDLSGKFLAE YQPEKLAAAE WHFETS-SHA PLILFGTLE- 275
               **. . : : ::: :: . . :   ::  :: ** **  .:.  *  **** *  .:**. : * .: ***  :

Q-loop
Ec       278  EEETNKFAIQ IPYALGIIAT RSVDTPVIGL KELMVQHEER IRNGMKAYSL LEQLRSGSTD QAVRDQFNSM 347
Gt       276  EDNEVKYALE IPYALSILAH NHPAAVVTGL NDIPEDERPP LY-------- ---------- ---------- 317
              *::  *:*:: *****.*:*  .   : * ** :::  :..   :

                             Q-loop              |  L |
Ec       348  KKDLGYGLLL KRYTPNVADA TEAQIQQATK DSIPRVAPLY FAFRIMVACG FLLLAIIALS FWS-VIRNRI 416
Gt       318  ---------- ---------- ---------- --------IH YLFDVMVTIG VFLMVVAAVY WLGSIFRWKW 349
                                                       :: : * :**: * .:*:.: *:  : . ::* :

Ec       417  GEKKWLLRAA LYGIPLPWIA VEAGWFVAEY GRQPWAIGEV LPTAVANSSL TAGDLIFSMV LICGLYTLFL 486
Gt       350  TAKNWFFGLL VAGGPLAMIA IEAGWYLAEV GRQPWILRGY MKTAEG--AT TSAHVDTMLV LFCLLYIVLV 417
                *:*::    : * **. ** :****::**  ***** :    : ** .  :  *:..:   :* *:* ** :::

Ec       487  VAELFLMFKF ARLGPSSLKT GRYHFEQSST TTQPAR 522
Gt       418  IASATVLIRM FRRNP----- VERELEERAN RGEVAP 448
              :*.  ::::: * .*       . .:*: :.   : *
```

Alignment of CydB

```
Ec         1  MIDYEVLRFI WWLLVGVLLI GFAVTDGFDM GVGMLTR--- FLGRNDTERR IMINSIAPHW DGNQVWLITA 67
Gt         1  ----MTLEVI GISVLWLFLF GYIIVASIDF GAGFFSVYSH WANQQHILHR IIQRYLSPVW EVTNVFLVFF 66
                   .*..*    :: ::*: *: :. .:*: *.*:::     : .::.  :* *: . ::* * : .:*:*:

Ec        68  GGALFAAWPM VYAAAFSGFY VAMILVLASL FFRPVGFDYR SKIEETRWRN MWDWGIFIGS FVPP---LVI 134
Gt        67  FVGIVGFFPK TAYYYGSILL VPASIAIVLL AIRGSYYAFH TYGETERNWY LLAYG-LTGL FIPASLSIVL 135
                .:.. :*  .     * :  *.  :.:. *  :*   : :: :  *  *    :  :* : *  *:*.   :*:

Ec       135  GVAFGNLLQG VPFNVDEYLR LYYTGNFFQL LNPFGLLAGV VSVGMIITQG ATYLQMRTVG ELHLRTRATA 204
Gt       136  TISEGGFVEE SAAGVALDYG KLFAS----- --PLSWSVVL LSVTSVLYIS AVFLTYYADA AGDEQARALL 198
               :: *.:::   . .*        ::.        *:.  . : :**  ::  . *.:*   : .   . ::**

Ec       205  QVAALVTLVC FALAGVWVMY GIDGYVVKST MDHYAASNPL NKEVVREAGA WLVNFNNTPI LWAIPALGVV 274
Gt       199  RRYALLWSGP TMLSALLIIY QLR----YHN PEHYDN---- ---------- ---LWN---V AWMLVISFLF 244
              :  **:       *:.: ::*  :       .  :**                      :*   :  * :    :.

Ec       275  LPLLTILTAR MDKAAWAFVF SSLTLACIIL TAGIAMFPFV MPSSTMMNAS LTMWDATSSQ LTLNVMTWVA 344
Gt       245  FVITVWLLGR QRRFGWAFIA LLFQYAFAFY AYGISHYPYL LYPYLTIYDG FTN-ETMAMA LIVAFIAGLL 313
              : : . * .*   : .***:    :  *  :  : **: :*:: : .   :  . :*  :: :   * : .:: :

Ec       345  VVLVPIILLY TAWCYWKMFG RITKEDIERN THSLY 379
Gt       314  LLIPSLYLLM RLFLFNKAYV KGKWEGGKG- ----- 342
              ::: .: **    : : * :  : . *. :
```

Alignment of CydX

```
Ec        1  MWYFAWILGT LLACSFGVIT ALALEHVESG KAGQEDI 37
Gt        1  MQTFLIMYAP MVVVALSVVA AFWVG-LKDV HVNE--- 33
             *  *  : .. ::. ::.*:: *: :  ::.  :..:
```

B) Alignment of the Q-loop sequence

```
Ec      249  QKTKLAAIE AEWETQPAPA AFTLFGIPDQ EEETNKFAIQ IPYALGIIAT RSVDTPVIGL KELMVQHEER 317
Gt      249  QPEKLAAAE WHFETS-SHA PLILFGTLEE DNEV-KYALE IPYALSILAH NHPAAVVTGL NDIPEDERPP 315
             *  **** *  .:**. : * .: ***  :: ::*. *:*:: *****.*:*  .   : * ** :::  :..

Ec      318  IRNGMKAYSL LEQLRSGSTD QAVRDQFNSM KKDLGYGLLL KRYTPNVADA TEAQIQQATK DSI 380
Gt      316  L--------- ---------- ---------- ---------- ---------- ---------- --- 317
             :
```

C) Alignment of *E. coli* CydA and CydB

```
CydA      1  MLDIVELSRL QFALTAMYHF LFVPLTLGMA FLLAIMETVY VLSGKQIYKD MTKFWGKLFG INFALGVATG 70
CydB      1  --------MI DYEVLRFIWW LLVGVLL--- ---------- ---------- ---------- --IGFAVTDG 27
                      : :: :  :  : *:* : *                                       :.:.*: *

CydA     71  LTMEFQFGTN WSYYSHYVGD IFGAPLAIEG LMAFFLESTF VGLFFFGWDR LGKVQHMCVT WLVALGSNLS 140
CydB     28  FDMGVGMLTR FLGRNDTERR IMINSIAPH- ---------- -------WDG N-------QV WLITAGGALF  72
             : * . : *. :   ..     *:  .:* .                    **           . **:: *. *

CydA    141  ALWILVANGW MQNPIASDFN FETMRMEMVS FSELVLNPVA QVKFVHTVAS GYVTGAMFIL GISAWYMLKG 210
CydB     73  AAWPMVYAAA FSG------- --FYVAMILV LASLFFRPVG FDYRSKIEET RWRN--MWDW GIFIGSFVPP 131
             * * :*  .  :..               ::  ::.*.:.**.      :   :  : .  *:   **    ::

CydA    211  RDFAFAKRSF AIAASFGMAA VLSVIVLGDE SGYEMGDVQK TKLAAIEAEW ETQPAPAAFT LFGIPDQEEE 280
CydB    132  LVIGVAFGNL LQGVPFNVDE YLRLYYTGN- ---------- ---------- ---------- ---------- 160
               :..*  .:   ...*.:    * :   *:

CydA    281  TNKFAIQIPY ALGIIATRSV DTPVIGLKEL MVQHEERIRN GMKAYSLLEQ LRSGSTDQAV RDQFNSMKKD 350
CydB    161  ----FFQLLN PFGLLAG--- ---VVSVGMI ITQGATYLQM RTVG-ELHLR TRATAQVAAL VTLVCFALAG 219
                  :*:   .:*::*        *:.:  : :.*    ::     . .*  :  *: :   *:    .     .

CydA    351  LGYGLLLKRY TPNVADATEA QIQQATKDSI PRVAPLYFAF RIMVACGFLL LAIIALSFWS VIRNRIGEKK 420
CydB    220  VWVMYGIDGY VVKSTMDHYA ASNPLNKEVV REAGAWLVNF NNTPILWAIP ALGVVLPLLT ILTARMDKAA 289
             :     :. * . : :    *   :  .*: :  ....  . * .       :     :.*.: : ::  *:.:

CydA    421  WLLRAALYGI PLPWIAVEAG WFVAEYGRQP WAIGEVLPTA VANSSLTAGD LIFSMVLICG LYTLFLVAEL 490
CydB    290  WAF--VFSSL TLACIILTAG IAMFPFVMPS STMMNASLTM WDATSSQLTL NVMTWVAVVL VPIILLYTAW 357
             * :  .: .: .*. * : **   :  :   .  :: :.  *     :*       ::: * :   :  ::* :

CydA    491  FLMFKFARLG PSSLKTGRYH FEQSSTTTQP AR 522
CydB    358  CYWKMFGRIT KEDIERNTHS LY-------- -- 379
                  *.*:   ..:: . :  :
```

Supplementary Figure 4 | Sequence comparisons of *bd* oxidase. A) Comparison between CydA, CydB and CydX/S from *E. coli* and *G. thermodenitrificans,* Ec: *E. coli*; Gt: *G. thermodenitrificans*; *, identical; :, conserved substitutions; ., semi-conserved substitutions; L, linker; Q-loop, amino acids contained within the *E. coli* Q-loop region; red letters, *E. coli* residues 263 to 302 not resolved in the structure, most likely due to their high flexibility, B) comparison between the Q-loop sequence from *E. coli* and *G. thermodenitrificans* and C) comparison between *E. coli* CydA and CydB. The sequence alignment was generated by ClustalW (www.genome.jp/tools-bin/clustalw).

A) B)

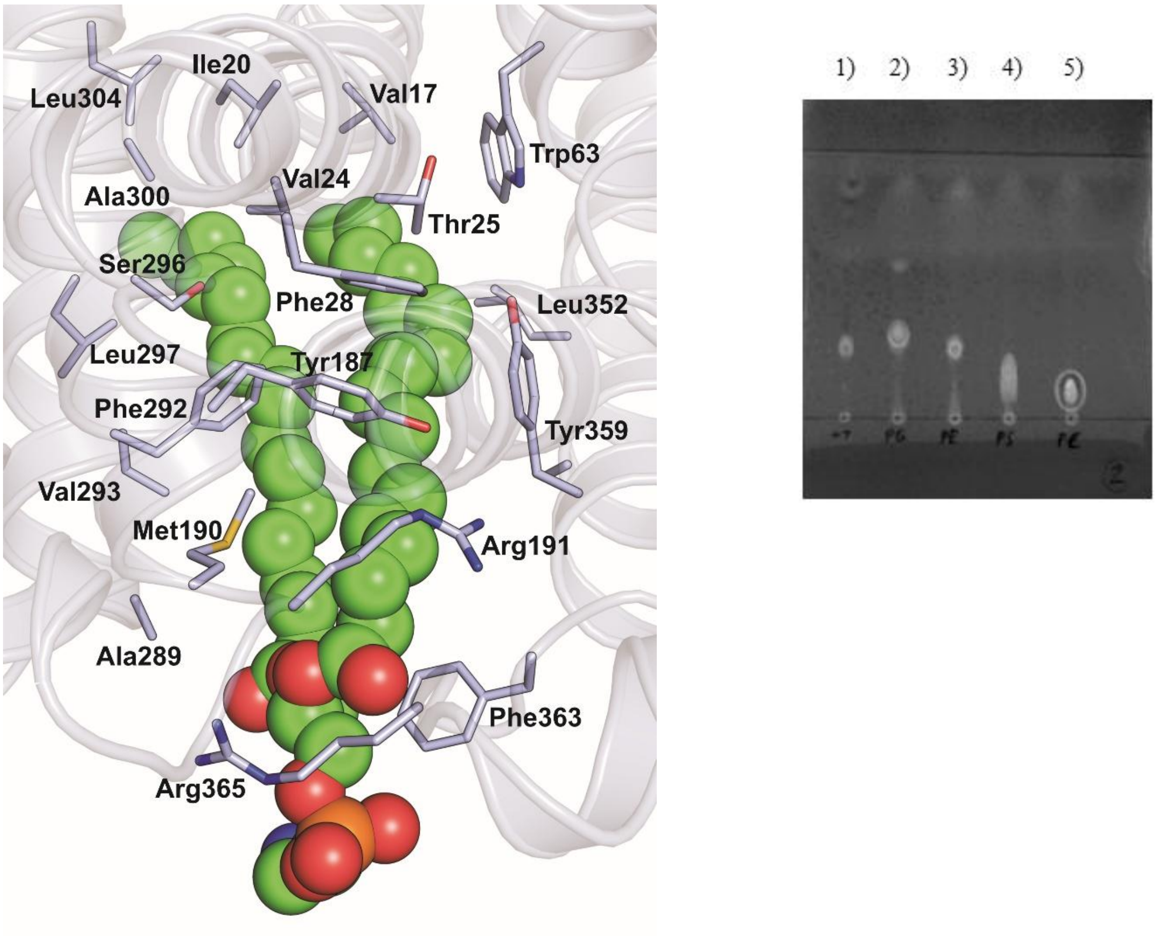

Supplementary Figure 5 | Distearyl glycerophosphoethanolamine is bound by numerous amino acid side chains of CydB. A) At the cytoplasmic side (bottom) Arg365 binds to the phosphate group. An alternative head group binding site may be provided by Arg191. B) The polar head group was identified by TLC ethanolamine. Lane 1: Extract from sample, 2: phosphatidylglycerol, 3: phosphatidylethanolamine, 4: phosphatidylserine and 5: phosphatidylcholine.

A)

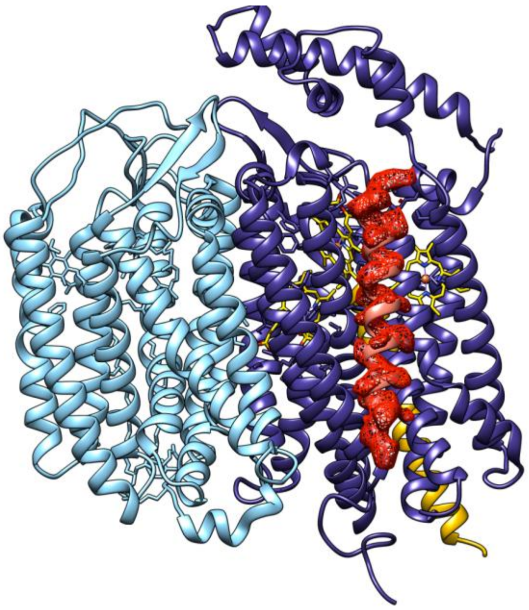

B)

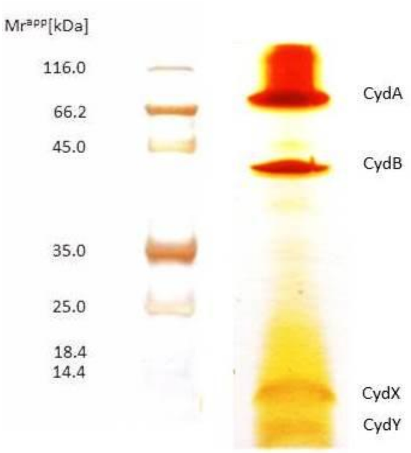

C)

```
ECOLI       1 MSTDLKFSLV TTIIVLGLIV AVGLTAALH- 29

SALTY       1 MSTDLKFSLI TTLIVLGVIV AGGLTAALH- 29
EDWTA       1 MESDLKYSLV TTVVALSLIV VAGLIVALH- 29
PHOPR       1 MEADLKFALI TTGVVFAILI GFGLTAIGA- 29
ENT38       1 MSTDLKYSLI TTVIVLSLIV FGGLTAALH- 29
ENTCL       1 MSTDLKYSLF TTVIVLSVIV FGALTAALH- 29
CITCO       1 MSTDLKFSLV TTIIVLGLIV AGGLTAALN- 29
KLEPN       1 MDTNLKFSLI TTIIALGVIV AFSLTAILH- 29
PANSA       1 MSTDLKFSLL TTVGALLMII AFSFTAILH- 29
SERPR       1 MDTDLKMSLF TTVCALAVII AFSFTAALN-
VIBHA       1 MEHDLKSALL IVVTIFAVLL SFGIIAITTA 30
              *. :.* :*   .:  : :::   .:
```

Supplementary Figure 6 | Presence of YnhF in the preparation and in the structure of *E. coli bd* oxidase. A) An unidentified membrane protein was seen as weak density of a TM helix (red mesh) in front of the portal towards the heme b_{595}. This blocks access of dioxygen to this heme. CydA is shown in dark blue, CydB in light blue and CydX in orange. The positions of the heme groups are indicated. B) The preparation of *E. coli bd* oxidase contains a fourth protein of the expected molecular mass of about 3 kDa. C) Sequence alignment of YnhF and homologous proteins. The residues within the *E. coli* sequence shown in red were found by mass spectrometry of the corresponding band from the gel identifying the protein as YnhF. We propose to re-name YnhF to CydY. The sequence alignment was generated by ClustalW (www.genome.jp/tools-bin/clustalw) using the same symbols as in Supplementary Fig. 4. Organism codes are from UniProtKB/Swiss-Prot (www.uniprot.org/docs/speclist).

(A)

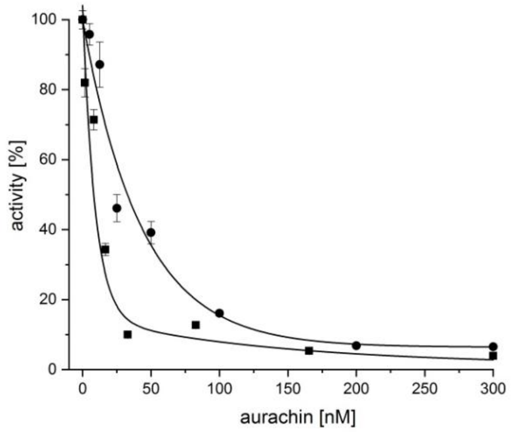

(B)

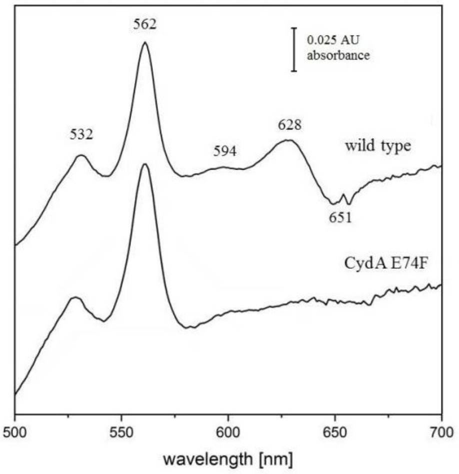

Supplementary Figure 7 | Inhibition of *bd* oxidase by aurachin C and D and UV/vis difference spectrum of the E74F^{A} variant. (A) Inhibition A of the duroquinol: dioxygen oxidoreductase activity of *bd* oxidase by aurachin C (filled squares) and D (filled circles). The apparent IC_{50} values derived from the curve are 12 and 35 nM, respectively. Activities were determined from three biological replicates. Error bars represent standard deviations. The error bars are equivalent throughout the figure. (B) UV-vis (dithionite-reduced) minus (air-oxidized) difference spectrum of a detergent extract of membranes from the parental strain and the E74F^{A} mutant. The conserved E74^{A} is located at the interface between CydA and CydB at the level of heme d in the *E. coli bd* oxidase. The extract from the mutant is lacking the absorbances of heme d at 628 and 651 nm, while the absorbance of heme b_{595} is slightly reduced and perturbed. Attempts to purify the *bd* variant from the mutant strain failed due to a disintegration of the complex.

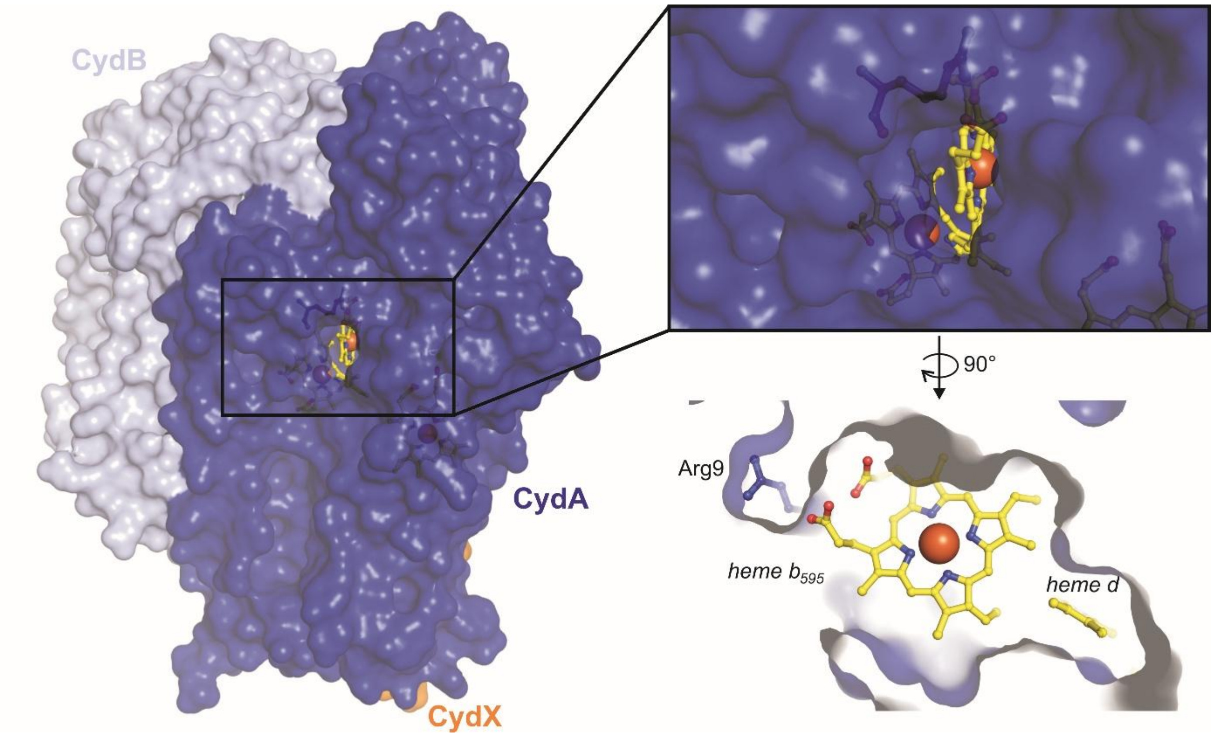

Supplementary Figure 8 | Conserved substrate channel toward b_{595}. Left: View on solvent accessible surface of *bd* oxidase disclosing the substrate channel in CydA leading to heme b_{595}. Top right: Close-up view on the channel entrance, the entrance features a positive charge, provided by Arg9. Bottom right: cross-section through the substrate channel to b_{595}. The cavity extends towards heme d, buried deeper inside subunit CydA but not accessible for dioxygen.

A) HisTrap ff

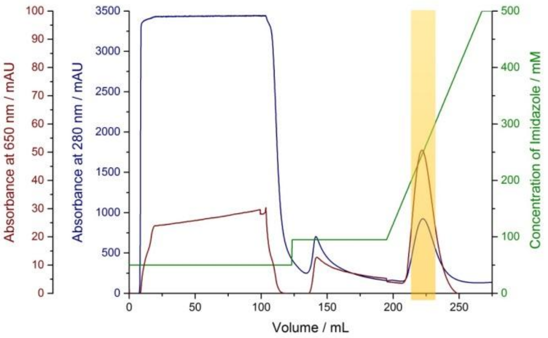

C) Superose 6 10/300 GL

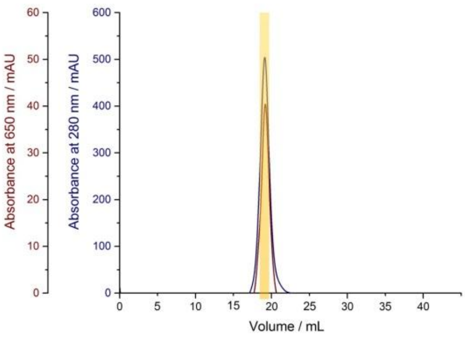

B) MonoQ 10/100 GL

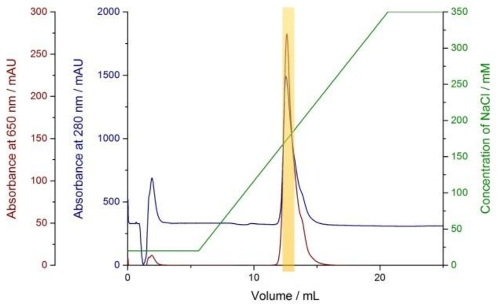

D) SDS-PAGE

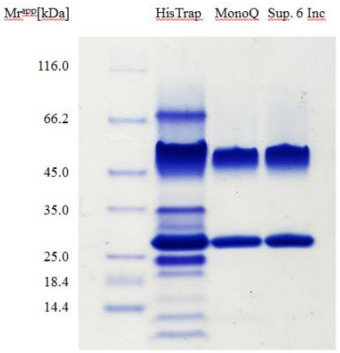

Supplementary Figure 9 | Preparation of *bd* oxidase from strain *E. coli* BL21* Δ*cyo*/ pET28a *cydA$_h$BX*. The elution profiles from A) HisTrap FF, B) MonoQ 10/100 GL and C) Sepharose 6 10/300 GL are shown. The absorbance at 280 nm is shown in blue, the absorbance at 650 nm in red and any ion gradients in green. The pooled fractions are indicated by the yellow area. D) SDS-PAGE of the preparation. On the left lane, the bands of the protein standard are marked by their individual apparent masses. The pattern of the pooled fractions from the HisTrap FF, the MonoQ 10/100 GL and the Sepharose 6 10/300 GL column are shown in the lanes from left to right.

Supplementary Table 1 | Cryo-EM parameters and statistics.

Data collection:	
Voltage (kV)	300
electron dose ($e^-/Å^2$)	59
magnification	75,000
recording mode	counting
movie frames	47
defocus range (µm)	1.4-2.2
number of collected movies	8663
Map parameters:	
Final particles (no.)	197,805
symmetry imposed	C1
Map resolution (Å)	3.3
FSC threshold	0.143
Map resolution range (Relion local res.) (Å)	3.22-3.84
Map sharpening B factor ($Å^2$)	-88
Model refinement:	
Initial model used (PDB code)	5DOQ
Model composition	
Non-hydrogen atoms	7347
Protein residues	910
Ligands	5
B factors ($Å^2$)	
Protein	61
Ligand	57
Validation:	
MolProbity score	1.60
Clashscore	5.49
Poor rotamers (%)	0.14
R.m.s. deviations	
Bond lengths (Å)	0.008
Bond angles (°)	1.354
Ramachandran plot	
Favored (%)	95.67
Allowed (%)	4.33
Outliers (%)	0.00

Supplementary Table 2 | Sequences of mutagenic primer. Exchanged nucleotides, bold; silent mutations, blue; italics, newly generated restriction sites.

Oligonucleotide	Sequence 5´-3´
cydB_A82F_fwd	CTGGCCGATGGTCTATGCC*GCA**TTC***TTCTCCGGCTTCTATGTGGCG
cydB_A82F_rev	CGCCACATAGAAGCCGGAGAA***GAA**TGC*GGCATAGACCATCGGCCAG
cydB_A137F_fwd	CGCCGCTGGTAATTGGTGTA**TTC**TTCGGTAACCTGTTGCAGGGC
cydB_A137F_rev	GCCCTGCAACAGGTTACCGAA**GAA**TACACCAATTACCAGCGGCG
cydB_A172F_fwd	GCTTAACCCGTTCGGCCTGCTG**TTC**GGCGTGGTGAGCGTAGGGATG
cydB_A172F_rev	CATCCCTACGCTCACCACGCC**GAA**CAGCAGGCCGAACGGGTTAAGC
cydA_E74F_fwd	GTGGCTACCGGTCTGACCATG**TTC**TTCCAGTTCGGGACTAACTGG
cydA_E74F_rev	GTTAGTCCCGAACTGGAA**GAA**CATGGTCAGACCGGTAGCCAC
